# TEAM-UP for quality: a cluster randomized controlled trial protocol focused on preventing pressure ulcers through repositioning frequency and precipitating factors

**DOI:** 10.1186/s12877-018-0744-0

**Published:** 2018-02-20

**Authors:** Tracey L. Yap, Susan M. Kennerly, Susan D. Horn, Nancy Bergstrom, Santanu Datta, Cathleen Colon-Emeric

**Affiliations:** 1 School of Nursing, Duke University, Durham, NC USA; 2 College of Nursing, East Carolina University, Greenville, NC USA; 3 School of Medicine, University of Utah, Salt Lake City, Utah USA; 4 School of Nursing, University of Texas Health Science Center at Houston, Houston, TX USA; 5 Department of General Internal Medicine, Duke University, Durham, NC USA; 6 Division of Geriatrics, School of Medicine, Duke University, Durham, NC USA

**Keywords:** Pressure ulcer, Pressure injury, Prevention, Nursing, Repositioning

## Abstract

**Background:**

Pressure ulcers/injuries (PrUs), a critical concern for nursing homes (NH), are responsible for chronic wounds, amputations, septic infections, and premature deaths. PrUs occur most commonly in older adults and NH residence is a risk factor for their development, with at least one of every nine U.S. NH residents experiencing a PrU and many NHs having high incidence and prevalence rates, in some instances well over 20%. PrU direct treatment costs are greater than prevention costs, making prevention-focused protocols critical. Current PrU prevention protocols recommend repositioning residents at moderate, high, and severe risk every 2 h. The advent of visco-elastic (VE) high-density foam support-surfaces over the past decade may now make it possible to extend the repositioning interval to every 3 or 4 h without increasing PrU development. The TEAM-UP (Turn Everyone And Move for Ulcer Prevention) study aims to determine: 1) whether repositioning interval can be extended for NH residents without compromising PrU incidence and 2) how changes in medical severity interact with changes in risk level and repositioning schedule to predict PrU development.

**Methods:**

In this proposed cluster randomized study, 9 NHs will be randomly assigned to one of three repositioning intervals (2, 3, or 4 h) for a 4-week period. Each enrolled site will use a single NH-wide repositioning interval as the standard of care for residents at low, moderate, and high risk of PrU development (*N* = 951) meeting the following criteria: minimum 3-day stay, without PrUs, no adhesive allergy, and using VE support surfaces (mattresses). An FDA-cleared patient monitoring system that records position/movement of these residents via individual wireless sensors will be used to visually cue staff when residents need repositioning and document compliance with repositioning protocols.

**Discussion:**

This study will advance knowledge about repositioning frequency and clinically assessed PrU risk level in relation to PrU incidence and medical severity. Outcomes of this research will contribute to future guidelines for more precise preventive nursing practices and refinement of PrU prevention guidelines.

**Trial registration:**

Clinical Trial Registration: NCT02996331.

## Background

Pressure ulcer/injury (PrU) prevention remains a challenge within the nursing home (NH) environment [1, 2]. A PrU is any skin lesion over a bony prominence resulting from prolonged exposure to pressure that causes capillary occlusion and eventual tissue necrosis. Most PrUs are avoidable, but those that do develop are associated with complications (e.g., chronic wounds, amputations, septic infections, and premature deaths) [3] and with overall deterioration in prognosis, compromising both a patient’s health status and quality of life. Estimated annual treatment costs ($9.1–11.6 billion in the US [4]) are greater than prevention costs [4–8], even without considering other costs to the individual and healthcare system (outpatient visits, work loss, and lawsuits), thus making prevention a priority [9].

Residence in a NH puts individuals at risk of developing PrUs [8], and the limited mobility prevalent among NH residents [10] increases the intensity and duration of pressure exposure – two factors leading to PrU development [11]. The severity of illness, including signs, symptoms, physiologic parameters, and disease factors, also affects the body’s response to pressure exposure and possibly increases risk for PrU development. A universally accepted approach to PrU prevention is to minimize pressure exposure through frequent moving/repositioning (hereafter referred to as repositioning) [12, 13] for residents clinically assessed as at-risk (typically Braden Scale© score ≤ 18).

Over the last decade, NH PrU incidence rates have declined from 11% [14] to 7.8% [15]. This decline can be attributed to the advent of viscoelastic (VE) high-density foam support surfaces that reduce pressure intensity by redistributing point pressure. VE surfaces make longer intervals between repositioning possible for at-risk residents confined to bed or chair [16]. However, in order to further improve PrU prevention approaches [17], we must determine 1) how frequently repositioning needs to occur in order to prevent PrU development, and 2) how residents’ medical severity (severity of illness), risk level (clinically assessed risk determined by the Braden Scale©), and repositioning frequency affect PrU development individually or in combination. It is not known whether a 2, 3, or 4 h time interval for repositioning of residents is most effective in preventing PrUs.

Current PrU prevention repositioning protocols (derived from two small 1962 studies [18]) recommend repositioning residents at moderate, high, and severe-risk every 2 h. With VE surfaces, it may be possible to reduce repositioning frequency by extending the repositioning interval (time elapsed between scheduled repositioning of residents) from 2 to 3 or even 4 h without an increase in PrU development. In the last decade, only two studies have examined repositioning protocols. One study reported fewer PrUs in residents using VE surfaces with a 4-h repositioning interval than in residents who used non-VE surfaces with more frequent repositioning [19, 20]. The second study, Turning for Ulcer ReductioN (TURN) trial, found no significant differences in PrU incidence between 2, 3, or 4-h repositioning intervals in a sample of moderate and high-risk residents using VE surfaces. In these two studies, 90% of all new PrUs occurred within the first 15 days [20]. However, new PrUs cannot be attributed to any single factor [21], and these analyses did not comprehensively examine changes in residents’ PrU risk levels or their physiological and disease factors (medical severity).

We will examine the effects of repositioning frequency (using assigned repositioning intervals of 2, 3, or 4 h) on residents at low, moderate, and high risk of developing PrUs (Braden Scale score 10-23) and will explore the effects of risk level and medical severity individually and in combination over a 4-week intervention period. Residents deemed to be at low risk are not commonly studied, but they too develop PrUs [2, 14–16] and are therefore included in this study. An FDA-cleared patient monitoring (PM) system using individual wireless tri-axial accelerometer sensors will record resident position and movement, automate repositioning schedules, provide visual cues to nursing staff [22] via electronic display screens at the nursing station and unit hallway(s) that indicate when each resident needs repositioning, and confirms completion of repositioning. This system provides a timestamp that repositioning occurred and addresses concerns about nursing electronic medical record (EMR) documentation compliance.

The specific aims of the TEAM-UP (Turn Everyone And Move for Ulcer Prevention) study are to:

- Aim 1: Determine differences in the incidence of new pressure ulcers (PrU) in nursing home (NH) residents at low, moderate, and high risk using VE support surfaces and repositioned at intervals of 2, 3, or 4 h, in nine randomly assigned NHs over a 4-week period, using the patient monitoring (PM) system to determine movement.
- Aim 2: Determine how medical severity components (as measured by a modified Comprehensive Severity Index [23, 24]), changes in clinically assessed risk level (low, moderate, high as measured by the Braden Scale© [25]), assigned repositioning intervals, and their interactions are associated with development of PrUs, controlling for resident demographics.
- Exploratory Aim: Evaluate PrU prevention intervention approaches in NH groups repositioned at 2, 3, or 4-h by: 1) comparing the intervention resource costs (VE support surfaces, PM system service/sensor use, fixed and variable labor costs for training and repositioning) and incremental cost-effectiveness ratio of cost per % reduction in PrUs, and 2) exploring staff and resident satisfaction with intervention approach.

### Significance

PrUs occur most commonly in older adults [6], and NH residence is a risk factor [8] for development. At least one of every nine U.S. NH residents will experience a PrU [14, 26] and many NHs have high incidence and prevalence rates, in some instances well over 20% [27, 28]. The estimated cost of treating PrUs ranges from $21,000–152,000 per PrU [29]. Effective prevention can reduce PrU incidence and avoid treatment costs while improving resident satisfaction, safety, and quality of life. Residents identified as moderate- or high-risk (who comprised 22–28% and 56–57% respectively of the NH population in two recent studies [12, 30]) are considered most likely to develop PrUs; however, low-risk NH residents also develop PrUs [19, 28, 31] and must be considered in any comprehensive prevention approach. PrUs are challenging to address, given the variations in NH size, staffing, and resident diversity, but creating cost-effective solutions is imperative because1 in every 5 adult Americans (some 72 million) will be > 65 years old by 2030 and PrUs could increase exponentially [6].

PrUs remain a multifactorial problem, although the advent of innovative products (e.g., VE support surfaces) has minimized some risk. In order to further reduce PrU incidence, it is important to both 1) test and challenge current prevention protocols to identify a gold standard, and 2) understand the intersection of multiple contributing factors to PrUs to identify additional opportunities for improvement. Improving prevention protocols is critical on multiple levels (i.e., individual, department, facility, society) given the cost of treating PrUs, the direct impact on both staff and residents, and the fact that overall costs of delivering prevention protocols is less than treatment costs.

The TEAM-UP study challenges the idea that repositioning must be completed every 2 h to best prevent the onset of PrUs. It builds on prior evidence from an intervention study [19, 20] showing that repositioning intervals may be extended from every 2 h to every 3 or 4 h without increasing PrU incidence, and attempts to expand the evidence base for PrU prevention by determining how residents’ medical severity, clinically assessed PrU risk level, and assigned repositioning interval affect PrU development individually or in combination. TEAM-UP extends previous study periods [19, 20] to 4-weeks (28 days) to help determine the optimal resident repositioning interval, and will: 1) place all NH residents on VE (high-density foam) support surfaces to avoid point pressure and redistribute pressure, so as to enable longer periods between repositioning; 2) enroll residents at low risk for PrU development in addition to those identified as moderate or high risk; 3) use a PM system with individual wireless sensors to track repositioning compliance with time-stamped documentation and provide repositioning reminders, while also providing automatic feedback for individual nursing staff and the team to improve communication; and 4) monitor fluctuations in resident characteristics and risk levels, which may be associated with development of PrUs. The study intervention will be carried out by NH staff in real-life practice conditions with protocol and training support, rather than by an external intervention team. The impact of the intervention on the occupational subculture of the nursing staff will be explored, as will the perspectives of both nursing staff and residents. Nurse champions will be trained to support staff as resources for protocol implementation and the use of the PM system and sensors.

## Methods

The TEAM-UP study is a cluster randomized clinical trial with facility-level use of an assigned repositioning interval as the primary intervention and will be conducted at 9 NHs that are all using VE support surfaces. Each participating NH will be randomly assigned to one of three intervention arms. Each arm will have 3 NHs with each NH using a single, NH-wide repositioning interval at either 2, 3, or 4 h as standard of care during the 4-week intervention period. Randomization will occur at the facility level. The sequence in which the repositioning intervals are to be implemented will be randomly ordered and, then assigned to NHs inclusive of each arm of NHs. Thus, 3 clusters of 3 NHs are created with randomly sequenced repositioning intervals.

**Fig. 1:**
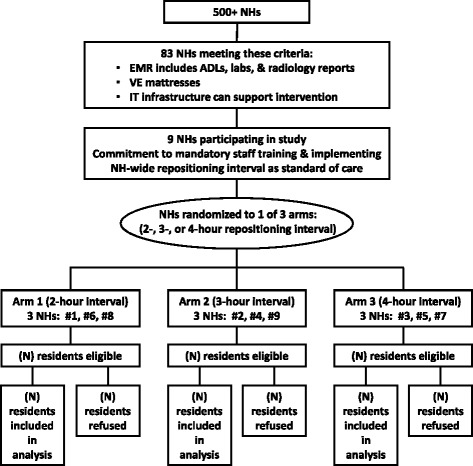
Flowchart for study protocol manuscript

A secondary cluster is created within each NH as the NH implements its standard risk assessment protocol; residents will be clinically categorized forming clusters by level of risk for developing a new PrU. Level of risk categorization into 3 groups is defined by a Braden score of low (15–18), moderate (13–14), or high (10–12) [32]. All eligible residents will wear a wireless triaxial accelerometer sensor throughout the intervention as part of the NH’s standard of care. Residents may refuse the sensor and can decline to move when prompted by staff as part of their right to refuse care (see Fig. 1). This pragmatic approach offers a unique opportunity to study a single NH-wide repositioning schedule, thus avoiding the potential for staff confusion associated with resident-level random assignment to repositioning intervals, which would require staff to simultaneously execute multiple turning schedules.

Medical severity components and clinically assessed risk level will be extracted from the EMR and examined for assessment-to-assessment change, thus allowing identification of medical severity components associated with development of a new PrU. We will use intervention resource cost elements (VE surfaces, PM system and sensor service, fixed and variable labor costs for training and repositioning) to assess implementation cost and calculate a cost-effectiveness ratio per % PrU reduction. NH nursing staff will be asked to complete a nursing culture assessment web-based survey administered electronically at their NH pre- and post-intervention. Focus groups will be conducted using semi-structured questions framed according to the Diffusion of Innovation model to gather/assess NH staff and resident perspectives of each group’s satisfaction regarding the repositioning interval and challenges experienced in use of the PM system.

Additional information is available on the study website (https://teamup.nursing.duke.edu).

We are funded to conduct this study from 07/01/2016 to 03/31/2021. We have completed implementation in 3 of the 9 nursing homes and additional study data collection and analyses will be completed by 03/31/2021.

### Sample, setting, recruitment

#### NH sample

Researchers established a collaboration with a United States, for-profit, Medicare-certified, intermediate and skilled NH system with > 450 NHs in over 30 states. A convenience sample of 9 NHs will be selected from volunteer facilities within the system if they have > 100 beds and are using viable VE mattresses examined 2 months prior to intervention implementation, and have full EMR use that includes activity of daily living (ADL) documentation and laboratory and radiology electronic results. Participating NHs must agree to implement the patient monitoring system technology with a NH-wide repositioning protocol, and agree to a mandatory nursing staff in-service training provided by the research team. Full-time and part-time nursing staff (RNs, LPNs, CNAs) working clinically with residents will participate in an NH-mandated education session to explain the study protocol and prepare them regarding PM system use. NH’s will be assigned in random order to a repositioning interval.

The NH will give all its residents/family an information sheet that explains the study and NH-wide adopted repositioning protocol and informs them of their right to refuse care and/or request a more frequent repositioning interval. The information sheet will also explain risks, benefits, and what is involved in participation.

#### Resident sample

All residents (≥ 18 years old) using a VE mattress (support surface) who are without PrUs and adhesive allergy and are clinically assessed as low, moderate, or high risk for new PrU development will participate in their respective NH-wide repositioning frequency protocol and receive standard PrU prevention care.

NH resident participants will include those residents at the time of study initiation and any residents newly admitted during the 4-week study period. All individuals who are NH residents for a minimum of 3 days and meet inclusion criteria will be included in data analyses, because superficial PrUs have been reported to occur within several hours to days following NH admission, and “deep tissue injury”, a type of PrU resulting from pressure on muscle and fascia is thought to appear on the surface of the skin approximately 3 days following exposure to pressure.

A waiver of informed consent has been obtained from the Primary Investigator’s (PI) Institutional Review Board. Data on newly admitted residents with less than a 3-day stay will not be included in the analysis, because deep tissue injuries that result from pressure prior to admission may not be visible for up to 3 days after pressure exposure. Residents will also be excluded from the analysis if PrUs are present at baseline, PrU risk is severe (Braden score ≤ 9), or if the resident is cared for on a non-VE specialty surface, has “do not turn orders” present, or has an allergy to the adhesive used on the sensor. Residents assessed as being at severe risk of developing a PrU (estimated ≤ 10% per NH) will be excluded because they typically require specialty surfaces and individualized and frequent repositioning, and their health status is generally more unstable. Data on all other residents will be included, using an intention-to-treat approach. The target N for analysis is 951; we aim to enroll 1100 residents at baseline. Residents who are discharged, transferred, or die will be included in the intention-to-treat analysis.

#### Staff sample for survey

(*N* = 1200) All nursing staff (registered nurses, licensed practical nurses, and certified nursing assistants) working clinically full- or part-time with residents will be eligible irrespective of job category at each NH. Will be asked to complete an anonymous web-based electronic survey via touch screen tablet.

#### Focus group(s) sample

Two focus group sessions (one of residents and one of staff; six participants/ group), will be conducted at three selected NHs (one NH per arm) to evaluate implementation and perspectives regarding approach. Using convenience sampling, English-speaking intervention participants will be eligible to join, irrespective of gender, race, ethnicity, and physical ability.

1. *Residents.* Residents (aged > 18) who are cognitively aware and willing to participate will be selected and consented to participate in one focus group per selected NH (total of three groups; *n* = 18 residents). Participants may have refused sensor monitoring.
2. *Staff*. Nursing staff (aged > 18) working clinically full- or part-time with residents will be selected irrespective of job category and consented for one focus group per selected NH (3 groups; *n* = 18 nursing staff).

### Risk and challenges

This study design includes NHs with the same corporate management to ensure that the same standard-of-care protocols are in place in each NH. A major challenge for NH research is the potential for staff turnover. Using a successful strategy from our prior studies [31], we secured a written commitment that the study will continue even if there is a change in one or more of corporate administrative leaders for the NH system. We also have designed the study to be robust to staff turnover and potential staff resistance to a new repositioning schedule, by incorporating mandatory training with supplemental on-site instruction as needed and also incorporating training into the NH’s new staff orientations. Another potential challenge is staff inconsistency in implementing the repositioning protocol standard-of-care; consistency in repositioning is important to being able to subsequently demonstrate that the repositioning at the assigned interval actually occurred [33]. In fact, in practice, extended repositioning intervals already are thought to be extended beyond 2 h [34, 35] and there are longstanding concerns that nursing staff documentation may be unreliable for use as evidence of repositioning [20, 31, 36, 37]. The PM system monitoring capabilities will enable concerns about repositioning protocol compliance and documentation to be addressed via time-stamped tracking of identified position changes.

### The intervention

Elements of the intervention include a NH-wide assigned repositioning interval 2, 3, or 4-h) with repositioning of residents using viable VE mattresses. Monitoring and documentation of repositioning intervention will be accomplished with use of a PM system. Table 1 describes the protocol components in detail.

**Table 1:** Protocol for implementation of intervention approach

Protocol Components	Rationale/Outcome	Persons Responsible	Time (Fig. 4)
(1) PrU Prevention and Study Protocol Education Content: Introduces all NH nursing staff to PrU etiology, evidence-based PrU prevention practices, benefits of repositioning, & EMR documentation. Outlines roles & responsibilities, staff work flow, & performance & documentation of movement of residents. Content includes NH-wide repositioning strategy & suggestions for best practice implementation with cueing every 2, 3, or 4 h (24 h/day, 7 days/wk) depending on NH’s hourly protocol. Delivery Strategies: • Researcher leads initial session containing videos embedded in PowerPoint overview with simulation of repositioning & documentation process, followed by questions & answers. • Reference cards provided to all staff summarizing PrU etiology & staff responsibilities in relation to study protocol, day-to-day procedures, & directions for carrying out the protocol & completing study measures & documentation.	Establish knowledge of PrU etiology prevention practices, & study protocol. Facilitate consistency in response to intervention protocols, safety, & group effectiveness. Encourage problem-centered thinking & learning by demonstration and return demonstration.	RNs, LPNs, NAs, NPs	Initial session week prior to repositioning protocol start; for new staff orientation: one 60-min session followed by one-on-one training as needed when working.
(2) Champion Training Champion Training Class: Champions will learn to facilitate the educational component & practice mentoring on study strategy, problem-solving about protocol implementation, EMR documentation, & observation checklist completion; Leaf sensor placement with system activation check & skin monitoring. Support by Research Facilitators: A researcher will contact each champion every week via phone or videoconferencing during the intervention for support, advising, or refreshers as needed; champions will also have a phone number to call to seek help from research staff as needed.	Prepare champions to mentor staff, build trust, maintain consistency, and facilitate sustained implementation.	Volunteer nursing staff	Week prior to repositioning protocol start: one 60-min session.
(3) Patient Monitoring System Training Focus is on patient monitoring, responding to the 2-, 3-, or 4-h visual cues on the screen displays located at nursing station and in hallway on clinical unit, and review sample data feedback & practice interpretation. Placement of Sensor on Resident: Focus is on licensed nurse & process for sensor placement and oversight of CNA routine observation. Staff will learn to place sensor and ensure its activation and to remove and discharge sensor. Leaf placement will be periodically checked by champions to ensure fidelity.	Staff will be prepared to monitor the screen display for repositioning status (cue & next repositioning times) and to complete the appropriate documentation.	RNs, LPNs, NAs, NPs	Week prior to repositioning protocol start: Part of PrU Prevention Protocol & Education session followed by one-on-one on-the-job training as needed.
(4) VE Support Surface Audit This will be completed at each NH site according to NH system’s policy. Each NH will provide audit documentation demonstrating that all mattresses are adequate and have been examined (those that were not adequate were replaced) within the last year according to NH system’s Mattress and Safety Audit Guidelines.	Ensure viable mattresses in use prior to intervention start	Research team and NH staff	Minimum of 1 month prior to repositioning protocol start.

#### Repositioning protocol

Residents who are in bed will be repositioned according to the predetermined NH-wide assigned repositioning interval of every 2, 3, or 4 h, and rotated from side to back to side. In accordance with International PrU prevention guidelines [13], residents will be positioned in the lateral position at no more than a 30^0^ tilt. While in the supine position, the head of the bed may be elevated during feeding, but the duration of elevation greater than 30^0^ will be limited to avoid exposure to friction and shear. Residents will wear pressure reduction boots if ordered by the NH; however, because there is no evidence that these perform better than elevating the heels using a pillow, the latter will be standard procedure in this study. According to NH policy, there is a standing order that nursing staff should assist non-bedfast residents while in the chair to stand/move/reposition each hour to relieve ischial pressure, preventive seating cushions will be used as necessary according to NH guidelines. PrUs resulting from sitting will appear on ischial tuberosities; these PrUs are clearly different from PrUs on the sacrum/coccyx, trochanter, and heels that occur when lying in bed. Repositioning devices (e.g., turning sheets or trapeze) will be used as appropriate to reduce friction and shear, and pillows and foam wedges will be used to maintain position according to international guidelines.

**Fig. 2:**
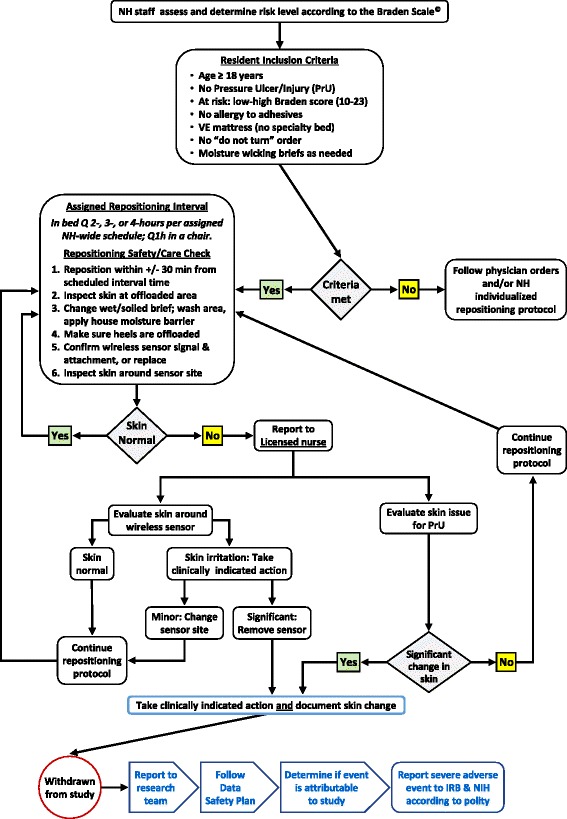
Repositioning and skin safety decision tree

In NHs, up to 9% of long-stay residents spend the majority of most days in bed or in a chair in their room [38]. Bates-Jensen et al. found that most residents spent 17 h a day in bed [34]. We expect, however, that 90% of residents will be sitting for at least part of the day. Repositioning will occur during the time that residents are in bed. Figure 2 is a visual depicting the required assessment and repositioning safety and skin care check to be performed by nursing staff and the criteria that must be met at each decision point in implementing the repositioning protocol.

#### VE support surface

All NH’s VE support surfaces (mattresses) in this study will meet or exceed international guidelines [39] for the minimum acceptable requirements for density and support to ensure that a resident’s mattress is flexible enough to deflect pressure over bony areas that come in contact with the mattress. VE surfaces will be examined 2 months prior to each NH’s intervention and all mattresses that are not viable will be replaced prior to study implementation.

#### The patient monitoring (PM) system

A wireless system for monitoring resident position and movement will be used to ***visually*** cue nursing staff on electronic display screens to ensure protocol adherence and to evaluate NH-repositioning protocol delivery fidelity [40]. This system is used in hospitals and NHs to enable caregivers to implement individualized repositioning protocols and it drives compliance with the care plan by visually notifying staff when repositioning is required [7, 41, 42]. Using a skin-sensitive medical grade adhesive backing (similar to an EKG electrode), a wireless, wearable single-patient use sensor will be placed by nursing staff on the resident’s upper chest beneath the clavicle on either the right or left side at approximately the midclavicular line (at least 4 in. away from a pacemaker or implantable cardioverter-defibrillator). The individual sensor has no on- or off-switch and activates automatically when the opaque backing is removed to expose the sensor to light. Each sensor will be associated with a specific resident, will have individualized repositioning parameters such as the 2-, 3-, or 4-h interval, and will track and indicate changes in body position. The PM system’s turn management software displays each resident’s repositioning history and current positional status in an organized and simple way on strategically placed display screens at the nursing station and mid-point in each hallway, making staff aware when repositioning actions are becoming necessary or are overdue.

### Treatment fidelity

Our treatment fidelity protocols use the National Institutes of Health Behavior Change Consortium’s [43] model. To ensure intervention integrity, repositioning of residents will be assigned to and performed by nursing staff. Each resident’s repositioning activity will be monitored with a wireless sensor and documented within the PM system, and randomly scheduled weekly observation will be conducted of staff turning/repositioning selected residents (6 per week x a minimum of 4 weeks) to provide a fidelity check of the system’s accurate detection of turning/repositioning activities. Studies and clinical data show that the device effectively detects position changes according to threshold parameters, like degree or angle of position change [44, 45]. Each NH will use a single NH-wide repositioning interval as the standard of care for low-, moderate-, and high-risk residents. This pragmatic approach offers a unique opportunity to study a single NH’s facility-wide repositioning schedule, thus avoiding the potential for staff confusion if more than one schedule is executed within a facility. Furthermore, each repositioning interval will be tested in three NHs using VE surfaces, enabling replication of findings.

### Data collection & storage (see Fig. 3 and Table 2)

#### Quantitative data collection procedures

##### NH-level data.

NH Company has agreed to provide each participating NH’s characteristics, including bed size and nursing staff hours, facility-level data (PrU period prevalence and incidence, staff number, mix, and turnover) at baseline (12 months prior) and during the intervention period. Staff mix, wage, and fringe benefit rates will be also be obtained. Data will be collected from publicly available sources (https://www.medicare.gov/nursinghomecompare/search.html). These data will be used as covariates in the multivariate analyses.

**Fig. 3:**
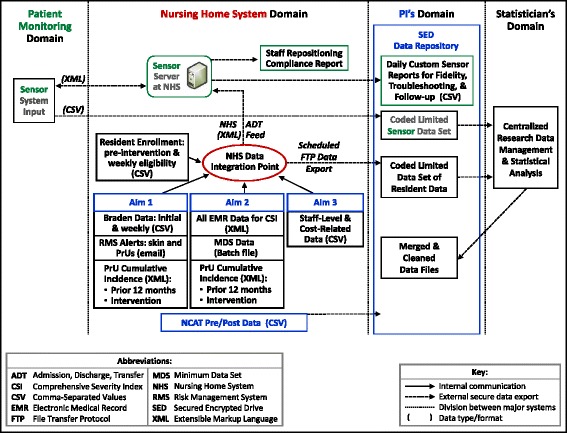
TEAM UP study high-level components diagram and key data sources

**Table 2:** Summary of concepts and instruments / measurements used to collect data

Concept Measured	Measures / Instrumentation	Source (Fig. 3)
Cumulative incidence of (new) PrUs for pre-intervention (baseline) period	Cumulative Incidence rate = [(# of residents with 1 or more new PrUs during a 1-month period) ÷ (# of NH residents with 3 or more day stay)] × 100; calculated monthly to establish baseline over the 12-month period before each NH intervention begins.	• MDS 3.0 • NH self-report • EMR data extract
Cumulative incidence of (new) PrUs for intervention period	Cumulative Incidence rate = [(# of residents with 1 or more new PrUs during intervention period) ÷ (# of residents participating in intervention for 3 or more days)] × 100; calculated for the full 4-week intervention period.	• MDS 3.0 • NH self-report • EMR data extract
Braden Scale©	The Braden scale is being used per protocol by participating NHs to assess resident risk for PrUs at intervention start and weekly thereafter.	• EMR data extract
Repositioning frequency	The scheduled duration of time for a resident’s pressure exposure as reflected by the 2-, 3-, or 4-h time interval at which the resident is repositioned; each NH will be randomly assigned to one of the study’s 3 arms and will use a single NH-wide repositioning frequency.	• PMS Central Server containing Monitoring Management Software
Recorded repositioning frequency	The actual time interval at which repositioning occurs, as documented by the Leaf sensor; recorded in clock time hours and minutes, 24 h per day, from start to end of resident’s participation in the 4-week intervention. Repositioning activities of residents will be captured using the Patient Monitoring System; Admission, Discharge, Transfer (ADT) feed from NHS to Monitoring System enables a match for Study ID# to resident movement, which is required for resident tracking in the event of room change, discharge/readmission, or other events.	• PMS Central Server containing Monitoring Management Software
Repositioning protocol adherence: % agreement	Percentage of agreement between scheduled repositioning frequency and repositioning frequency recorded by wireless sensor: [(scheduled clock time - recorded clock time = # minutes difference) ÷ 60] × 100]; calculated for each resident’s repositioning episode.	• PMS Central Server containing Monitoring Management Software
Medical Severity —Comprehensive Severity Index (CSI)	Calculated score on severity resulting from distillation of physiologic parameters, signs, symptoms, laboratory results, physical findings, and diagnoses, using the modified Comprehensive Severity Index (CSI), a risk adjustment system. The more abnormal the signs and symptoms, the higher the severity score: Level 1 is normal to mild, and Level 4 is catastrophic, life-threatening, or likely to result in organ failure. Individual components thought to influence a resident’s skin tolerance to pressure exposure will be monitored from assessment to assessment for Leaf, resident care parameters, CSI, and Braden score, with data extracted from EMR and the most recent MDS 3.0 assessment (initial, annual, quarterly, or change in status).	• MDS 3.0 • EMR data extract
Clinically assessed risk level (Braden Scale)	Total summed score (range 6 to 23) on the *Braden Scale for Predicting Pressure Sore Risk©*, comprised of 6 subscales (sensory perception, mobility, activity, moisture, nutrition, and friction & shear). Braden Score will be categorized as low (15–23), moderate (13–14), or high (10–12) risk. Collected weekly during intervention. Routinely collected by NH on admission, weekly × 4, quarterly, and upon suspected change in health condition. Change in category will be extracted.	• EMR data extract
Culture Assessment	Demographic & NCAT data will be used to assess the basic characteristics of participants and healthcare setting and clinical area worked. Data to be collected from each participant include, Length of time employed, job category, age, and gender.	• Qualtrics survey on iPads
Staff mix	The number of RNs, LPNs, and CNAs who work at each NH per day each day during the 4-week intervention at that NH, and for 12 months prior to intervention start.	• NH self-report
Staff turnover rate	[(Number of nursing staff (CNA, LPN, & RN) who leave during the time period) ÷ (Number of nursing staff at beginning + Number of nursing staff at the end ÷ 2)] × 100.	• NH self-report
Labor cost: 1) Training 2) Conducting repositions	1) Time needed for training nursing staff multiplied by respective wage & fringe rates of training participants, 2) number of repositioning’s for each NH and study arm and time to conduct repositioning collected by wireless sensor system; multiplied by nurse wage & fringe rate.	• NH self-report
Non-labor cost inputs	Market prices paid fully depreciated over their respective useful life will be used to calculate daily equipment cost rates. Includes VE surfaces, Leaf services, and sensor use.	• NH self-report

##### Resident-level data.

Resident data extracted from the PM system database and the 12-month baseline and 4-week intervention MDS (batch file) and EMR; each resident will be assigned a study ID number prior to data extraction. New PrU incidence will be collected during the intervention period. Baseline data will be collected for 12 months just prior to intervention launch and will consist of EMR, MDS, and PrU incidence (Table 2) data. Intervention data extraction (of EMR, MDS, and PM system data) will occur weekly during the intervention 4-week period. The PM system will schedule resident repositioning frequency and gather resident repositioning sensor data automatically according to NH standards of care. Data collected by each resident’s sensor will be automatically communicated wirelessly through a proprietary mesh network of relay antennas to the respective NH’s Central Monitoring Station containing turn management software. Wireless sensor data on all monitored residents will be stored by the PM system. The wireless sensor number will become associated with a resident study identifier to be created as described below. The study ID number will be used to facilitate data comparisons. Wireless sensor data for all monitored residents will be securely stored in the PM system database.

Bench testing and clinical trials of the PM system’s wireless sensor have demonstrated it to be a valid measure of active and passive movement (repositioning). The sensor’s sensitivity accuracy is +/− 2.5% consistent with the industry standard for a linear triaxial accelerometer. Studies and clinical data show that the device effectively detects position changes according to threshold parameters, like degree or angle of position change. Wireless sensor data on all monitored residents will be securely stored in the patient monitoring system database. Wireless sensor data along with other resident data, including EMR data, will be transferred to the researchers for analysis. Turn management software in the PM system displays each resident’s repositioning history and current positional status in an organized and simple way on strategically placed nursing station and hallway display screens, making staff aware when resident repositioning actions are becoming necessary or are overdue. The percentage of on-time repositioning will be calculated for the period during which the wireless sensor is worn.

##### EMR integration of patient monitoring system and CSI procedures.

The EMR will be used to facilitate data collection and export of study-related data, which includes an interface with wireless sensor data. This approach eliminates costly labor-intensive, in-person fidelity checks and minimizes research burden on staff. Individual wireless sensors will communicate with wireless antennas placed throughout the NH, permitting resident monitoring in all NH areas. The patient monitoring system will allow us to ascertain repositioning protocol adherence by measuring consistency between scheduled, performed, and documented repositioning frequency with random weekly observational repositioning checks to ensure repositioning fidelity. With CSI and other resident data, medical severity components will be assessed by staff based on NH policies and procedures and monitored in relation to risk level.

##### Staff-level data.

The web-based survey will be administered electronically on-site with touch screen tablet, pre/post the 4-week intervention during the time the employee is at work. The Nursing Culture Assessment Tool (NCAT) [46, 47] survey contains 19-items and there are some demographic items (i.e., length of time employed, job category, age, gender, education level, shift), and will be administered pre/post intervention. At the time of survey data collection, data will be transferred through a secured server from which it will be downloaded into a database for statistical analysis. Data will be stored on the study’s secure encrypted drive (SED).

### Measures

**Fig. 4:**
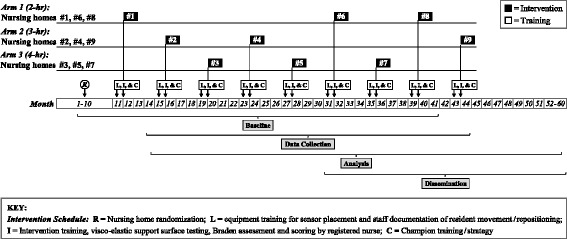
Study timeline and data collection schedule

The measures and time points at which data will be collected are summarized in Table 2, and Fig. 4 is the study timeline and data collection schedule.

#### Qualitative data collection procedures

##### Focus groups.

In three randomly selected NHs, one from each hourly repositioning protocol, focus group sessions (one for staff, one for residents) will be held to explore the intervention approach and implementation. There will be one group of NH staff (*n* = 6) and one group of NH residents (*n* = 6) per site. NH staff focus group discussion will be stimulated using semi-structured questions to gather participant perspectives on implementation challenges. Each session will last 30–45 min. At the start of all focus group sessions participants will be consented. All focus groups will be recorded and verbatim transcripts (with no participant identifiers) will be prepared for subsequent use in data analysis.

#### Analysis

##### Quantitative analyses.

We hypothesize that there will be no significant difference in PrU incidence between the 3 treatment arms in the study using three risk level groups: high risk (Braden Scale Score, 10–12), moderate risk (Braden Scale Score, 13–14), and low risk (Braden Scores > 14). The hypothesis of no group difference will be tested by examining whether the 95% confidence intervals of the rates of PrU and the 2-h repositioning overlap. If so, the hypothesis for no group difference will be confirmed. We will also determine how changes in medical severity components (as measured by a modified Comprehensive Severity Index), change in clinically assessed risk level (low, moderate, high as measured by the Braden Scale) and their interactions with repositioning schedule are associated with PrU development.

Descriptive statistics will be used to examine resident status, interventions, outcomes, and cost elements. For categorical variables, we will create contingency tables and use chi-squared or Fisher’s exact tests to determine the significance of bivariate differences. For continuous variables, we will use analysis of variance or non-parametric tests, depending on variable distributions. A two-sided *p*-value < 0.05 will be statistically significant. Intervention cost by NH and study arm and incremental cost-effectiveness ratios (ICERs) will be calculated and compared for cost per percentage reduction in PrUs. Sensitivity analysis will be conducted with the range of observed repositioning times, nurse salaries, and market prices for non-labor inputs to assess the robustness of the ICERs and their sensitivity to labor and non-labor input prices.

Because we will be examining PrU development from assessment to assessment, the rate of PrU incidence will be modeled as a binary outcome using repeated measures logistic regression and a specified working correlation matrix that models the correlation of responses within subjects. Full likelihood estimation methods are not appropriate for repeated measures logistic regression, so generalized estimating equations (GEEs) will be used instead with model fit assessed using the quasi-likelihood information criterion (QIC). Robust standard errors, clustered at the facility level, will be employed to account for covariance among residents within facilities over time. To test the robustness of models, we will also use non-linear mixed models using PROC GLIMMIX as another option for modeling the data (instead of GEEs using PROC GENMOD). With PROC GLIMMIX, we can use random effects to model the covariance rather than clustered standard errors. As predictors, we will include time-invariant resident characteristics, e.g., age, gender, Medicaid payer, and repositioning schedule, as well as time-varying characteristics, such as resident severity measure, risk level as determined by Braden Scale© score, staffing characteristics (e.g., number, mix, and turnover), and suggested MDS resident data elements as covariates. We may lag some time-varying resident characteristics by one assessment because we want to study the associations of repositioning time with PrU development, controlling for the starting point of the resident for that assessment interval. Interaction variables will be included in the predictor data set.

Residents without PrUs who drop out of the study after at least a three-day stay but before the conclusion of 4 weeks will be included in the analysis based on intent to treat. Residents who develop a Stage 3 or worse PrU, or deep purple discoloration, will be removed from the study per repositioning protocol, but will be considered a completed case if the ulcer developed on the coccyx, sacrum, trochanter or heel. Residents who develop ulcers on sites not thought to be influenced by bed position or limited mobility, or related to sitting, will continue to be followed for analysis, but will not be included in the NH-wide protocol.

##### Statistical power.

According to previous research, the average PrU incidence rates range from 5.2–15%. At the time of study design, the nine NHs within the NHS were identified with the highest PrU incidence rates which averaged 9.2% (SD = 3.3%). In power analyses, we used 3.5% for PrU incidence as the expected rate for intervention since it is the highest rate found in the TURN study (for moderate-risk patients). The PrU incidence rates have been translated into an odds ratio of 0.38 (= 3.5%/9.2%) as the effect size to detect. For Aim 1, odds ratios from a logistic regression of PrU incidence rates in the three treatment arms (i.e., at 2-, 3-, and 4-h repositioning frequencies), controlling for risk level, are proposed to test the difference in PrU incidence rates between the 4-h arm and the 2-h arm as well as between the 4-h arm and the 3-h arm at 4 weeks; we are assuming that the distance between the 2-h arm and the TURN 3-h odds ratio will be larger. A sample size of *N* = 951 (*n* = 317 for each arm) will be required to detect an odds ratio of .38 with a power of 0.95 at a one-tailed significance level of α = 0.05, according to G*Power. We are using a power of 0.95 instead of the conventional power of 0.80 to ensure that we will have a smaller Type II error to test the hypothesis of no-group difference that we expect to fail to reject. We are using a one-tailed test since we are only interested in determining whether the PrU rate is higher for assigned repositioning intervals longer than the current 2-h standard practice.. For Aim 2, GEE is proposed. A sample size of *n* = 114 for each group (total *N* = 342) will be required to detect an effect size of 5.7% (= 9.2% - 3.5%) with a power of 0.80 at a one-tailed significance level of α = 0.05, using the algorithms GEESIZE developed by Dahmen, et al., on an autoregressive structure for residual correlations [48]. Since the nine NHs will be randomly assigned to the three treatment arms, risk level will be the only potential covariate to be controlled in the models, and this is already considered in the power analyses. Therefore, we will target a sample size of *N* = 951 (the larger of the two required sample sizes) for both aims while taking into account potential losses due to turnover, attrition, and exclusion criteria. While this proposed study may look like a non-inferiority or equivalence trial, we will run regular one-tailed superiority tests, because (as noted earlier) we are only interested in examining whether or not the PrU rate is higher for less frequent repositioning than for the standard 2-h repositioning. (We assume that 2-h repositioning was followed to produce the 9.2% average PrU incidence rate across our 9 study NHs, because that is the current standard of care). Also, according to Snapinn [49], non-inferiority trials have many controversial assumptions and limitations that need to be made or addressed, and that is beyond the control of our study. For incidence, it is difficult to specify a non-inferiority margin for PrU rates. Instead, we will test the hypothesis of no group-difference by examining whether the 95% confidence intervals of the odds ratios (for Aim 1) or the PrU rates (for Aim 2) of the two pairs of compared groups (e.g., 4-h arm vs. 2-h arm and 4-h arm vs. 3-h arm) overlap. If the confidence intervals do overlap, the hypothesis for no-group difference will be confirmed.

##### Missing data.

When data are missing, we will make adjustments depending on the variable and its intended use in the analyses. Residents with missing data will be deleted from analyses and continuous variables with missing data collapsed into categorical data, and cases with missing information for the continuous variable placed into a category using corroborating data. For example, we may not always have a resident’s body mass index, but may have other weight- and height-related information (e.g., resident description as ‘emaciated’) that allows us to categorize a resident broadly, e.g., as underweight. We will use various types of imputation as appropriate and check assumptions about ‘missing at random’. To exclude unrealistic values and obvious outliers from the analysis, we will set ranges for some variables, and values beyond those ranges will be deemed improbable and excluded. These values may also be sent back to the NHs for correction.

##### Qualitative analysis.

NH staff and resident satisfaction with the intervention approach will be explored using semi-structured questions to staff and resident focus groups. A standardized topical guide will be used to evaluate 1) common perceptions related to satisfaction with the intervention approach, 2) areas of strength, and 3) areas for improvement. Verbatim transcripts will be checked against original recordings for accuracy. Data will be analyzed assisted by NVivo 11 along with field notes taken during the focus groups. As in the PI’s prior clinical trial, transcripts will be analyzed using thematic content analysis; matrices used to organize the data and category summaries will be compared across NHs and staff and resident groups. A directed approach will identify core concepts, and the analytic plan will include use of satisfaction-based a priori codes and allow for new themes to emerge post hoc. Analytical rigor will assure that 1) the study measures what is intended (credibility), 2) results are similar when the work is repeated in the same way, in the same context with the same participants and methods (dependability), 3) the findings are the results of the experiences of the participants (confirmability), and 4) the results are applicable to other situations (transferability).

## Discussion

This study will contribute to understanding of an optimal resident repositioning interval using VE surfaces for NH residents at different risk levels; further, the study will contribute to a much-needed understanding of the contribution of medical severity as a precipitating factor for PrU development. Effective prevention can potentially reduce PrU incidence and avoid treatment costs while improving overall resident safety and quality of life.

## Abbreviations

ICER: Incremental cost-effectiveness ratio
ID: Identification
MDS: Minimum Data Set
NCAT: Nursing culture assessment tool
NH: Nursing home
NHS: Nursing home system
PM system: Patient management system
PrU: Pressure ulcer/injury
RMS: Risk Management System
VE: Visco-elastic
